# A genetic screen identifies *C. elegans eif-3.H* and *hrpr-1* as pro-apoptotic genes and potential activators of *egl-1* expression

**DOI:** 10.17912/micropub.biology.001126

**Published:** 2024-02-16

**Authors:** Yanwen Jiang, Barbara Conradt

**Affiliations:** 1 Cell and Developmental Biology, University College London

## Abstract

During *C. elegans* development, 1090 somatic cells are generated of which 131 reproducibly die, many through apoptosis. The *C. elegans* BH3-only gene *egl-1* is the key activator of apoptosis in somatic tissues, and it is predominantly expressed in ‘cell death' lineages i.e. lineages in which apoptotic cell death occurs. *egl-1* expression is regulated at the transcriptional and post-transcriptional level. For example, we previously showed that the miR-35 and miR-58 families of miRNAs repress *egl-1* expression in mothers of ‘unwanted' cells by binding to the 3′ UTR of *egl-1* mRNA, thereby increasing *egl-1* mRNA turnover. In a screen for RNA-binding proteins with a role in the post-transcriptional control of *egl-1* expression, we identified EIF-3.H (ortholog of human eIF3H) and HRPR-1 (ortholog human hnRNP R/Q) as potential activators of *egl-1* expression. In addition, we demonstrate that the knockdown of the *eif-3.H* or *hrpr-1* gene by RNA-mediated interference (RNAi) results in the inappropriate survival of unwanted cells during *C. elegans* development. Our study provides novel insight into how *egl-1* expression is controlled to cause the reproducible pattern of cell death observed during *C. elegans* development.

**Figure 1. Genetic screen for activators of egl-1 expression using RNA-mediated interference:**
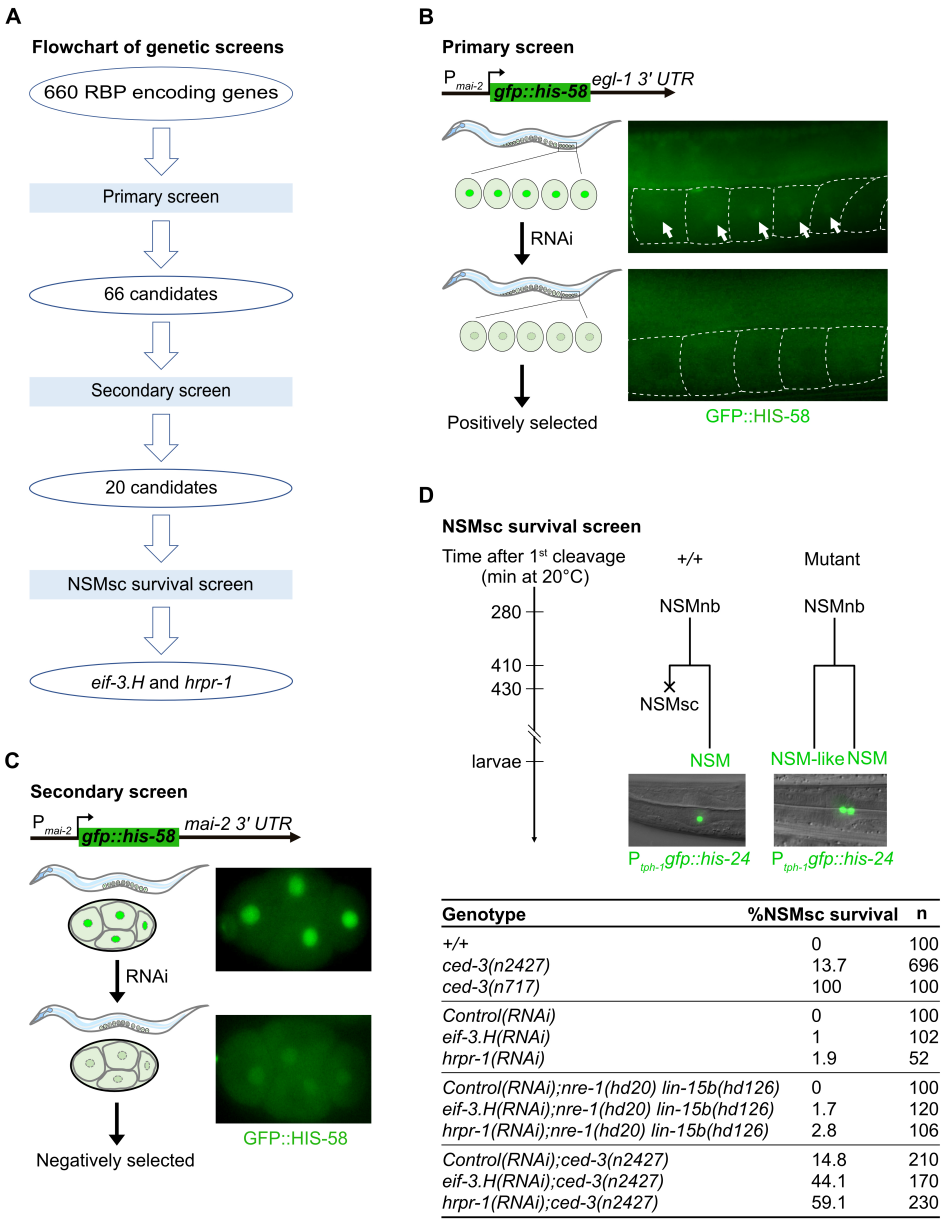
A) Flowchart of genetic screens for activators of *egl-1* expression. (B) Primary (positive) screen for activators of *egl-1* expression. After RNAi-mediated knockdown of RBP genes, candidates were identified by screening for a decrease in GFP:: HIS-58 signal in oocytes of animals carrying the reporter P *_mai-_*_2_ *gfp:: his-58 :: egl-1 3′ UTR* ( *bcSi26* ). White arrows point to GFP:: HIS-58 signal in oocyte nuclei. (C) Secondary (negative) screen. Nonspecific candidates were eliminated by screening for a decrease in GFP:: HIS-58 signal in embryos of animals carrying the reporter P *_mai-_*_2_ *gfp:: his-58 :: mai-2 3′ UTR* ( *bcSi25* ). (D) NSM sister cell (NSMsc) survival screen. (Top) Schematics showing the NSM lineage in wild-type ( *+/+* ) and *ced-3 (717)* animals (Ellis and Horvitz, 1986). The two bilaterally symmetric neurosecretory motoneuron (NSM) neuroblasts (NSMnb) (left and right) divide ~410 minutes after the first zygotic cleavage (at 20°C), each generating one NSM neuron, which is programmed to survive, and one NSM sister cell (NSMsc), which is programmed to die (‘unwanted' daughter cell) (Sulston et al., 1983). In wild-type ( *+/+* ) animals, the NSMsc undergoes apoptotic cell death, resulting in one NSM from each NSM neuroblast. When apoptosis is blocked, the NSMsc inappropriately survives, resulting in an extra ‘NSM-like' cell. The NSM and the ‘undead' NSMsc can be identified in the anterior pharynx of L3/L4 larvae using the reporter *P _tph-1_ gfp:: his-24* (Yan et al., 2013). (Bottom) RNAi knockdown of *eif-3.H* or *hrpr-1* causes NSMsc survival. To enhance RNAi efficiency in the NSM lineage, RNAi experiments were also performed in the *nre-1 ( hd20 ) lin-15b ( hd126 )* background (Schmitz et al., 2007). The percentage of NSMsc survival is enhanced in the background of *n2427* , a weak loss of function mutation of *ced-3* (Shaham et al., 1999). The sample size (n) is shown in the table. The complete genotypes of strains used are provided in Table 1.

## Description

Programmed cell death removes unwanted cells and helps shape organs during development (Suzanne and Steller, 2013) . Dysregulation of programmed cell death contributes to several diseases such as cancer, neurodegenerative or autoimmune diseases (Favaloro et al., 2012) . *Caenorhabditis elegans* ( *C. elegans* ) is a powerful model for studying programmed cell death. Programmed cell death during *C. elegans* development occurs in a highly reproducible pattern. Sulston and co-workers discovered that, among 1090 somatic cells generated during the development of a *C. elegans* hermaphrodite, precisely 131 cells die, many through apoptosis (Conradt et al., 2016; Horvitz, 1999; Sulston and Horvitz, 1977; Sulston et al., 1983) . The process of apoptosis is tightly regulated through a genetic pathway that is evolutionarily conserved from nematodes to mammals. In *C. elegans* , this pathway consists of four key components: *egl-1* , *ced-9* , *ced-4* , and *ced-3* (Conradt et al., 2016; Horvitz, 1999) . The *egl-1* gene is necessary and sufficient for apoptosis and encodes a pro-apoptotic BH3-only protein, EGL-1 , which binds to the anti-apoptotic Bcl-2-like protein CED-9 in unwanted cells. This displaces a dimer of the Apaf1-like protein CED-4 from CED-9 , thereby allowing CED-4 to form the apoptosome, which facilitates the autocatalytic activation of the CED-3 caspase. Activated CED-3 cleaves multiple substrates, ultimately leading to cell death. In contrast to *ced-9* , *ced-4* , and *ced-3* , which appear to be broadly expressed at least during *C. elegans* embryogenesis (Chen et al., 2000; Maurer et al., 2007) , *egl-1* expression is essentially restricted to cell death lineages (Conradt and Horvitz, 1999; Nehme et al., 2010) . Thus, the spatiotemporal pattern of *egl-1* expression and, hence, the control of *egl-1* expression is critical for the highly reproducible pattern of cell death observed during *C. elegans* development.

*egl-1* expression during *C. elegans* development is regulated at the transcriptional level by lineage-specific transcription factors that act through specific *cis* -acting elements upstream or downstream of the *egl-1* transcription unit (Conradt et al., 2016) . In addition, *egl-1* expression is controlled at the post-transcriptional level by miR-35 and miR-58 family miRNAs that act through the 3′ UTR of the *egl-1* mRNA to repress *egl-1* expression in mothers of unwanted cells, thereby preventing their precocious death (Sherrard et al., 2017) . Apart from binding sites for miR-35 and miR-58 family microRNAs, the *egl-1* 3' UTR contains additional conserved elements (Extended data figure 1). For this reason, we propose that factors other than microRNAs, such as RNA-binding proteins (RBPs), may contribute to the post-transcriptional regulation of *egl-1* expression and, hence, the highly reproducible pattern of cell death during *C. elegans* development.

To identify RBPs that promote *egl-1* expression, we performed a systematic RNAi (RNA-mediated interference) screen in *C. elegans* . To that end, we first generated a comprehensive list of previously reported *C. elegans* RBPs. An initial list of *C. elegans* RBP-encoding genes published by Wang et al. contains 319 genes that were identified by searching for genes encoding RNA-binding domains (RBDs) (Wang et al., 2009) . By searching for additional putative RBDs, Tamburino et al. increased the number of putative *C. elegans* RBP-encoding genes from 319 to 887 (Extended data table 1a) (Tamburino et al., 2013) . They included additional putative RBDs and protein classes such as dsRBDs and ribosomal proteins as well as C2H2 zinc finger- and SAM domain-containing proteins. In addition, systematic approaches were employed to experimentally map mRNA-binding proteins in yeast and mammalian cells by capturing *in vivo* cross-linked mRNA–protein complexes and by identifying associated proteins by mass spectrometry (Scherrer et al., 2010; Tsvetanova et al., 2010) . In a poly(A)-containing mRNA-capturing experiment, Matia-González et al. identified 594 proteins that interact with polyadenylated mRNAs in *C. elegans* (Matia-González et al., 2015). These mRNA-binding proteins are encoded by 591 genes (Extended data table 1b). However, only a small fraction of these 591 RBP genes (151) overlaps with the 887 RBP genes reported by Tamburino et al. (Extended data figure 2A). In addition, many previously reported RBPs, such as GLD-3 (Eckmann et al., 2002) , MEX-3 and PUF-8 (Ariz et al., 2009) , are missing from this list of 591 RBP genes, suggesting that the RBPs identified by Matia-González et al. do not represent all RBPs in *C. elegans* . Thus, we incorporated the lists published by Tamburino et al. and Matia-González et al. and conducted Gene Ontology (GO) and phenotype enrichment analyses (Extended data figure 2B, Extended data table 1c-1e). Genes with general functions, such as genes encoding tRNA-binding proteins or ribosomal subunits, were excluded (Extended data table 1d). Interestingly, some RBP genes are also enriched in phenotypes such as ‘cell death variants' (Extended data table 1e). These genes were retained in the final list for the RNAi screen. The final RBP compendium contained 800 genes (Extended data figure 2B, Extended data table 1f) of which 660 genes are represented in the Ahringer RNAi library (Kamath and Ahringer, 2003; Kamath et al., 2003) (Extended data table 1g). These 660 genes were subjected to the following RNAi screens for activators of *egl-1* expression (referred to as ‘ *egl-1* activators').

We first screened the 660 genes for potential *egl-1* activators using an *egl-1* 3′ UTR reporter ( Figure 1B, P rimary screen) (Sherrard et al., 2017) . In this reporter, the *egl-1* 3′ UTR is fused to a fusion of the coding sequences of *gfp* and Histone 2B gene *his-58* ( *gfp:: his-58* ), and the expression of the resulting fusion gene is driven by the promoter of the gene *mai-2* , which is ubiquitously transcribed (Ichikawa et al., 2006) . The use of the *mai-2* promoter ensures transcription of the reporter in all cells, which allows us to monitor the impact of the 3′ UTR on reporter expression. A single copy of this reporter was inserted into the *C. elegans* genome, generating the transgene P *_mai-2_ gfp:: his-58 :: egl-1 3′ UTR* ( *bcSi26* ) (Sherrard et al., 2017) . The expression of P *_mai-2_ gfp:: his-58 :: egl-1 3′ UTR* is repressed in embryos; however, in oocytes, moderate expression is detected ( Figure 1B ) (Sherrard et al., 2017) . By screening for a decrease in GFP:: HIS-58 signal in oocytes, 66 activator candidates were identified ( Figure 1A, 1B, Extended data table 2a). After the primary screen, we conducted a secondary (negative) screen for activators that are specific to the *egl-1* 3′ UTR. To that end, we used a single copy integration of the *mai-2* 3′ UTR reporter P *_mai-_*_2_ *gfp:: his-58 :: mai-2 3′ UTR* ( *bcSi25* ). This reporter differs from the *egl-1* 3′ UTR reporter ( *bcSi26* ) only in its 3′ UTR but it is ubiquitously expressed in all cells ( Figure 1C ) (Sherrard et al., 2017) . By screening for a decrease in GFP:: HIS-58 signal in embryos carrying P *_mai-_*_2_ *gfp:: his-58 :: mai-2 3′ UTR* ( *bcSi25* ), 41 out of 66 candidates were considered general nonspecific activators and were excluded from subsequent analyses. The remaining 25 candidates were considered specific for the *egl-1* 3′ UTR. The identities of the RNAi clones for these candidates were verified through Sanger sequencing. Twenty of them contained the correct insert ( Figure 1A, E xtended data table 2b).

The loss of activators of *egl-1* is expected to reduce *egl-1* activity and result in a cell-death defective (Ced) phenotype, namely, the inappropriate survival of unwanted cells (Conradt et al., 2016; Nehme and Conradt, 2008) . In wild-type embryos, the NSMsc dies soon after its birth ( Figure 1D ). When apoptosis is blocked, for example by a strong *ced-3* loss-of-function mutation *n717* (Ellis and Horvitz, 1986) , the NSMsc survives and forms an NSM-like cell. The NSM and ‘undead' NSMsc can be visualized by the expression of the reporter P *_tph-1_ gfp:: his-24* ( Figure 1D ) (Yan et al., 2013) . RNAi knockdown of *eif-3.H* or *hrpr-1* (also known as *hrp-2* ) caused a low rate of NSMsc survival in *bcSi126* (P *_tph-1_ gfp:: his-24* ) animals (1% for *eif-3.H (RNAi)* and 1.9% for *hrpr-1 (RNAi)* , respectively) ( Figure 1D ). Most *C. elegans* neurons are resistant to RNAi (Firnhaber and Hammarlund, 2013; Schmitz et al., 2007) . The *nre-1 ( hd20 ) lin-15b ( hd126 )* background has been shown to enhance RNAi efficiency in neurons (Schmitz et al., 2007) . For this reason, we also performed RNAi in a *nre-1 ( hd20 ) lin-15b ( hd126 )* background. In this background, RNAi knockdown of *eif-3.H* or *hrpr-1* caused 1.7% or 2.8% NSMsc survival, respectively ( Figure 1D ). By contrast, 0% NSMsc survival is detected in *bcSi126* (P *_tph-1_ gfp:: his-24* ) wild-type ( *+/+* ) or *bcSi126* (P *_tph-1_ gfp:: his-24* ); *nre-1 ( hd20 ) lin-15b ( hd126 )* animals that are fed with the control RNAi clone. We also determined NSMsc survival in the sensitized background of the weak *ced-3* loss-of-function mutation *n2427* (Shaham et al., 1999) . In *bcIs66* (P *_tph-1_ gfp:: his-24* ); *ced-3 ( n2427 )* animals, the apoptosis pathway is partially inactivated, resulting in 13.7% NSMsc survival ( Figure 1D ). In addition, *bcIs66* (P *_tph-1_ gfp:: his-24* ); *ced-3 ( n2427 )* animals fed with the control RNAi clone show 14.8% NSMsc survival. In contrast, *eif-3.H (RNAi)* and *hrpr-1 (RNAi)* show a significant enhancement of NSMsc survival in *bcIs66* (P *_tph-1_ gfp:: his-24* ); *ced-3 ( n2427 )* animals (44.1% for *eif-3.H (RNAi)* and 59.1% for *hrpr-1 (RNAi)* , respectively) ( Figure 1D ). These data suggest that *eif-3.H* and *hrpr-1* have pro-apoptotic activity and contribute to the activation of apoptosis possibly through promoting *egl-1* expression at the post-transcriptional level.

EIF-3.H is an ortholog of human eIF3H, which regulates the translation of mRNAs (Lee et al., 2015) . In zebrafish, it was shown that eIF3H promotes target gene translation during embryogenesis by targeting specific mRNAs to polysomes (Choudhuri et al., 2013) . In *C. elegans* , EIF-3.H was reported to promote axon guidance (Schmitz et al., 2007) . Our results show that *C. elegans* EIF-3.H possibly acts as an activator of *egl-1* expression. In the future, it will be interesting to determine whether EIF-3.H enhances *egl-1* mRNA translation by recruiting *egl-1* mRNA to polysomes. HRPR-1 (also known as HRP-2 ) is an ortholog of human hnRNP R, hnRNP Q (Syncrip) and ACF protein, the essential complementation factor in ApoB mRNA editing (Kinnaird et al., 2004) . *C. elegans* HRPR-1 contains three RNA-recognition motifs (RRM) and a C-terminal RG/RGG repeat element, indicating that it has RNA-binding activity (Kinnaird et al., 2004) . In addition *,* HRPR-1 regulates mRNA splicing by binding to UCUAUC splicing regulatory elements within target mRNAs, which include *unc-52* mRNA and *lin-10* mRNA (Kabat et al., 2009) . In mammals, hnRNP R regulates mRNA localization in neurons (Dombert et al., 2014; Glinka et al., 2010) . For example, hnRNP R directs the localization of *β-actin* mRNA to axons by binding to the 3′ UTR of *β-actin* mRNA (Glinka et al., 2010; Rossoll et al., 2003) . hnRNP Q has been reported to regulate mRNA splicing (Chen et al., 2008) as well as mRNA transport (McDermott et al., 2012) , translation (Svitkin et al., 2013) and stability (Grosset et al., 2000) . Therefore, *C. elegans* HRPR-1 may be involved in several aspects of the lifecycle of *egl-1* mRNA. In summary, our data suggest that, in addition to microRNAs, RBPs are likely to be involved in the control of *egl-1* expression at the post-transcriptional level and the activation of apoptosis during *C. elegans* development.

## Methods

***C. elegans* strains and maintenance**

The strains were maintained at 20°C on nematode growth medium (NGM) plates with *E. coli* OP50 bacterial lawns (Brenner, 1974) , unless otherwise specified. The stains used are listed in Table 1. The mutations and transgenes used were: LG I: *bcSi25* [P *_mai-2_ gfp:: his-58 :: mai-2 3ʹ UTR + unc-119 (+)* ] (Sherrard et al., 2017) , *bcSi26* [P *_mai-2_ gfp:: his-58 :: egl-1 3ʹ UTR + unc-119 (+)* ] (Sherrard et al., 2017) ; LG III: *bcIs66* [P *_tph-1_ gfp:: his-24 + lin-15 (+)* ] (Yan et al., 2013) , *bcSi126* [P *_tph-1_ gfp:: his-24 :: tbb-2 3′ UTR + unc-119 (+)* ] (This study), *unc-119 (ed3)* (Maduro and Pilgrim, 1995) ; LG IV: *ced-3 ( n2427 )* (Shaham et al., 1999) , *ced-3 ( n717 )* (Ellis and Horvitz, 1986) ; LG V: *ltIs44* [P *_pie-1_ mCherry::PH(PLC1 ^delta1^ ) + unc-119 (+)* ] (Audhya et al., 2005) ; LG X: *nre-1 ( hd20 )* (Schmitz et al., 2007) , *lin-15b ( hd126 )* (Schmitz et al., 2007) .

**Cloning and plasmid construction**

To construct the plasmid pBC1969 (P *_tph-1_ gfp:: his-24 :: tbb-2 3′ UTR* ), a 1.7 kb DNA fragment of the *tph-1* promoter region including the first exon was first amplified by PCR from *C. elegans* genomic DNA with primers 5ʹ-TGCATCGCGCGCACCGTACGTTCTCGCGAATTGCGGCCGAC-3ʹ and 5ʹ-GGAGCTGAAAGTACAGAAATTAC-3ʹ. Next, a 958 bp GFP fragment was amplified by PCR from the plasmid pBC1484 (Sherrard et al., 2017) with primers 5ʹ-ATTTCTGTACTTTCAGCTCCATGAGTAAAGGAGAAGAACTTTTC-3ʹ and 5ʹ-ACAACAGCGGAATCAGACATACTAGTTCTAGAGCGGCCGCCAC-3ʹ. Then, a 730 bp *his-24* fragment was amplified by PCR from *C. elegans* genomic DNA with primers 5ʹ-ATGTCTGATTCCGCTGTTGTTG-3ʹ and 5ʹ-TTAGGCCTTGGCGGCTGGCT-3ʹ. Finally, a 371 bp *tbb-2* 3 *′* UTR fragment was amplified by PCR from the plasmid pCFJ601 (Frokjaer-Jensen et al., 2012) with primers 5ʹ-AGCCAGCCGCCAAGGCCTAAATGCAAGATCCTTTCAAGCATTC-3ʹ and 5ʹ-AGAGGGTACCAGAGCTCACCTAGGTGAGACTTTTTTCTTGGCGGCAC-3ʹ. These fragments have 20 bp overlapping ends and were then assembled into the backbone pCFJ350 (Frokjaer-Jensen et al., 2012) between the *BsiWI* and *AvrII* restriction sites using the NEBuilder HiFi DNA Assembly Master Mix (NEB, #E2621L). The Phusion High-Fidelity DNA polymerase (NEB, #M0530L) was used for PCRs. The insert region of plasmid generated was confirmed by Sanger sequencing.

**Microinjection and transgenic animals**

To generate the Mos1 transposon-mediated Single-Copy Insertion (MosSCI) (Frokjaer-Jensen et al., 2012; Frokjaer-Jensen et al., 2008) transgene *bcSi126* [P *_tph-1_ gfp:: his-24 :: tbb-2 3′ UTR + unc-119 (+)* ], the universal MosSCI strain EG8080 [ *oxTi444* *unc-119 (ed3)* III] was used for germline microinjection and for targeted insertion onto chromosome III. The plasmid pBC1969 [P *_tph-1_ gfp:: his-24 :: tbb-2 3′ UTR + unc-119 (+)* ] was injected at a concentration of 30 ng/μl with co-injection plasmids 50 ng/μl pCFJ601 (P *_eft-3_ Mos1 transposase* ), 10 ng/μl pGH8 (P *_rab-3_ mCherry:: unc-54 3′ UTR* ), 2.5 ng/μl pCFJ90 (P *_myo-2_ mCherry:: unc-54 3′ UTR* ), and 5 ng/μl pCFJ104 (P *_myo-3_ mCherry:: unc-54 3′ UTR* ).

**Genetic screen by RNA-mediated interference**

Genetic screen by RNA-mediated interference (RNAi) was performed using the updated Ahringer RNAi feeding library (Kamath and Ahringer, 2003; Kamath et al., 2003) distributed by Source BioScience Ltd ( https://sourcebioscience.com ). This library covers ~87% of the currently annotated *C. elegans* protein-coding genes. Bacterial RNAi clones carrying the constructs that express relevant dsRNAs were cultured in 100 µL of LB medium containing 100 μg/mL carbenicillin in a 96-well plate at 37°C overnight. 10 µL of each bacteria culture was seeded into individual wells of a 12-well NGM plate containing 6 mM IPTG and 100 μg/mL carbenicillin as described previously (Rolland et al., 2019) . The seeded plates were incubated at 20°C overnight in the dark to induce dsRNA expression before use.

In the primary screen, the *egl-1* 3′ UTR reporter P *_mai-2_ gfp:: his-58 :: egl-1 3′ UTR* ( *bcSi26* ) was used to screen for a decrease in *gfp:: his-58* expression. Ten L3 larvae carrying P *_mai-2_ gfp:: his-58 :: egl-1 3′ UTR* ( *bcSi26* ) were transferred into each well of the 12-well NGM plate seeded with bacterial RNAi clones. After the animals were fed with bacterial RNAi clones for 48 hours, the expression of *gfp:: his-58* in nuclei of oocytes was scored. In wild-type animals, this reporter is repressed in embryos but moderately expressed in oocytes and germ cells. If *gfp:: his-58* expression was reduced in oocytes after the knockdown of an RBP gene, this RBP was considered an activator candidate of *egl-1* expression. In this screen, *gfp* RNAi and control RNAi were used as the positive control and negative control, respectively.

In the secondary (negative) screen, the *mai-2* 3′ UTR reporter P *_mai-2_ gfp:: his-58 :: mai-2 3′ UTR* ( *bcSi25* ) was used to exclude non-specific regulators by screening for a decrease in *gfp:: his-58* expression in 4-cell stage embryos. Candidates that reduced the expression of the *mai-2* 3′ UTR reporter after their knockdown were excluded. The identities of bacterial RNAi clones were confirmed by Sanger sequencing of the insert in the RNAi construct.

The percentage of NSMsc survival after RNAi-mediated knockdown of RBP genes was determined in the following way. Three L3 stage animals carrying the NSM reporter P *_tph-1_ gfp:: his-24* were transferred to NGM plates seeded with bacterial RNAi clones. After three days, L3/L4 stage F1 progenies were scored for extra NSM-like cells, which are found in the anterior pharynx and labelled by the reporter P *_tph-1_ gfp:: his-24* (Yan et al., 2013) . For RBP genes that cause larval arrest upon knockdown, L1/L2 stage F1 progeny was scored. In this screen, the control RNAi clone was used as a negative control.

## Reagents

**Table 1. List of strains used in this study.**

**Table:** 

**Strain**	**Genotype**	**Source**
**EG8080**	*oxTi444 unc-119(ed3)* III	CGC
**MD3203**	*bcIs66* III; *ced-3 ( n717 )* IV	(Yan et al., 2013)
**MD3437**	*bcIs66* III; *ltIs44* V	(Chakraborty et al., 2015)
**MD3712**	*bcIs66* III; *ced-3 ( n2427 )* IV; *ltIs44* V	(Chakraborty et al., 2015)
**MD3851**	*bcSi25* I; *unc-119(ed3)* III	(Sherrard et al., 2017)
**MD3852**	*bcSi26* I; *unc-119(ed3)* III	(Sherrard et al., 2017)
**MD4700**	*bcSi126 unc-119(ed3)* III	This study
**MD4704**	*bcSi126* *unc-119(ed3)* III; *nre-1 ( hd20 )* *lin-15b ( hd126 )* X	This study

## Extended Data

Description: List of RBP genes in C. elegans. Resource Type: Dataset. DOI: 10.22002/fjp7q-n1732

Description: egl-1 activator candidates. Resource Type: Dataset. DOI: 10.22002/cdeer-7ah33

Description: Conserved features in the egl-1 3' UTR. The C. elegans egl-1 3′ UTR contains conserved elements in comparison to those of three other Caenorhabditis species. (www.ebi.ac.uk/Tools/msa/clustalo). . Resource Type: Dataset. DOI: 10.22002/nhsx6-qv793

Description: Analysis of C. elegans RNA-binding proteins. (A) Overlap analysis of RNA-binding protein (RBP)-encoding genes reported by Tamburino et al., (2013) and Matia-Gonzalez et al., (2015). (B) Pipeline for RBP gene analyses, characterization, and selection. . Resource Type: Dataset. DOI: 10.22002/yspxf-ram42
